# Radiographic Thigh Muscle Measurements Are Independently Associated with 1-Year Mortality Following Hip Fracture Surgery

**DOI:** 10.2106/JBJS.OA.25.00256

**Published:** 2025-11-19

**Authors:** Duco W.P.M. Laane, Robert K. Wagner, Thirushan Wignakumar, Robin Hu, Marco Tulio Di Stefano, Fatmanur Aydin, Annemarie van Rossum du Chattel, Marcos R. González, Matthew Mariyampillai, Kihyun Kwon, Jacob Mandell, Detlef van der Velde, Michael J. Weaver

**Affiliations:** 1 Division of Orthopaedic Trauma, Department of Orthopedics, Brigham and Women's Hospital, Boston, Massachusetts; 2 Harvard Medical School Orthopaedic Trauma Initiative, Boston, Massachusetts; 3 Department of Surgery, St. Antonius Ziekenhuis, Utrecht, the Netherlands; 4 Department of Geriatrics, Universitair Medisch Centrum Utrecht, Utrecht, the Netherlands; 5 Division of Orthopaedic Trauma, Department of Orthopedics, Massachusetts General Hospital, Boston, Massachusetts; 6 Harvard Medical School, Boston, Massachusetts; 7 Division of Sports Medicine, Department of Orthopedics, Massachusetts General Hospital, Boston, Massachusetts; 8 Department of Radiology, Brigham and Women's Hospital, Boston, Massachusetts

## Abstract

**Background:**

Geriatric hip fractures remain associated with significant mortality and morbidity. Identifying factors associated with such outcomes is an important step for improvement of prognostication and supporting individualized care. Sarcopenia is a known risk factor for mortality and thigh muscle measurements on plain radiographs can serve as a proxy measure of sarcopenia. Therefore, the objective was to determine whether radiographic thigh muscle measurements were independently associated with 1-year mortality following hip fracture surgery.

**Methods:**

All consecutive patients aged 70 years or older undergoing operative treatment for an isolated hip fracture at 2 urban Level 1 trauma centers between 2018 and 2020 with preoperative radiographs displaying the distal-and-middle femur were included. Thigh muscle diameter and soft tissue size was measured on anteroposterior and lateral radiographs using standardized anatomical landmarks. Multivariable logistic regression was performed to determine if there were independent associations with 1-year mortality for the variables assessed. Inter-rater reliability for each measurement was evaluated by calculating intraclass correlation coefficients (ICCs).

**Results:**

One hundred ninety-nine patients (median age 85 years, 68% female) were included. One-year mortality was 22%. After adjusting for age, sex, smoking status, preinjury living situation, Charlson Comorbidity Index, frailty, and body mass index, a greater thigh muscle diameter on anteroposterior radiographs was associated with lower odds of 1-year mortality (adjusted odds ratio 0.74, 95% confidence interval 0.56-0.97, p = 0.028). There was no significant association with thigh muscle diameter on lateral radiographs or with total soft tissue diameter on anteroposterior or lateral radiographs. The ICCs demonstrated good-to-excellent reliability for all radiographic measurements.

**Conclusion:**

Greater thigh muscle diameter measured on anteroposterior radiographs was independently associated with decreased 1-year mortality following hip fracture surgery, with each centimeter increase in diameter being associated with an average reduction in odds of 26%. This finding should be interpreted in the context of the limited sample size, homogenous patient population, and range of observed thigh muscle sizes. Overall, these results suggest that radiographic parameters may potentially serve to complement currently used modalities, such as frailty assessment, in supporting individualized care.

**Level of Evidence:**

Prognostic Level III. See Instructions for Authors for a complete description of levels of evidence.

## Introduction

The number of hip fractures worldwide is projected to rise to 4.5 million by 2050 due to an increasingly aging population^1^. A hip fracture is a major life event for geriatric patients, with a 1-year mortality rate of 22% to 33% following surgery^2,3^. Identifying prognostic factors to complement currently used modalities, such as frailty assessment, can help improve prognostication, risk stratification, and support individualized care. Previous studies determined that factors associated with higher morbidity and mortality rates following a hip fracture include advanced age, more comorbidities, frailty, cognitive impairment, prior nursing home residence, impaired physical function, and sarcopenia^4-6^.

Sarcopenia is defined as the presence of low muscle strength, low muscle mass or low physical performance, and is an important driver for adverse outcomes after hip fractures^6-9^. As such, measures of sarcopenia may be useful for prognostication of outcomes. Sarcopenia can be determined using grip strength and gait speed tests, but these are not routinely performed in the acute hip fracture setting. Another commonly used methodology is to determine the psoas muscle area on abdominal computed tomography (CT) scans at the L3 and L4 vertebrae^10^. However, CT scans are not routinely obtained in the diagnostic workup for hip fractures.

More recently, Lurie et al. determined that thigh muscle measurements obtained from plain radiographs could predict psoas muscle area at L3 and L4 levels on CT and therefore predict sarcopenia^11^. Compared with CT, radiographs are inexpensive, widely available, and part of the routine diagnostic workup for hip fractures. Given that sarcopenia is a known risk factor of mortality, and that thigh muscle measurements can serve as a proxy for sarcopenia, these measurements may play a valuable role in the prognostication of outcomes for hip fracture patients.

Therefore, the primary objective of this study was to determine whether radiographic thigh muscle measurements were independently associated with 1-year mortality following hip fracture surgery.

## Methods

### Patients

All consecutive patients aged 70 years or older who underwent operative treatment of an isolated hip fracture at 2 urban Level 1 trauma centers between 2018 and 2020 were retrospectively identified from an institutional hip fracture database. This database registers all surgically treated hip fracture patients aged 70 years or older who present to the emergency department at one of the participating centers. Only patients aged 70 years or older are included in this database because these patients routinely undergo a Comprehensive Geriatric Assessment (CGA) at the participating centers. Patients were included if they underwent operative management of an isolated femoral neck, intertrochanteric or subtrochanteric fracture and received radiographic imaging of the distal-and-middle femur as part of the diagnostic workup. At the authors institutions, a CGA-based Frailty Index (CGA-FI) was developed and disseminated among clinical services between 2018 and 2019^12^. The CGA-FI is a validated measure of frailty and detects subtle health variations using a deficit accumulation model that considers issues across all CGA-domains^12-15^. As the CGA-FI was introduced during the study period, it was not collected for all eligible patients. Some patients underwent a CGA without a corresponding CGA-FI score, as completion depended on whether the attending geriatrician had been trained to calculate it. To enable the use of the CGA-FI as a confounder in statistical analysis, patients without CGA-FI data were excluded. Furthermore, patients were excluded if they sustained additional injuries beyond the hip fracture, if imaging was unavailable, or if imaging review revealed a fracture outside the inclusion criteria. This study obtained institutional review board (Protocol #: 2024P002691) and was performed in accordance with the ethical standards laid down in the 1964 Declaration of Helsinki and its later amendments. This article was prepared in accordance with the STROBE checklist (Appendix 1) ^16^.

### Radiographic Measurements

**Fig. 1:**
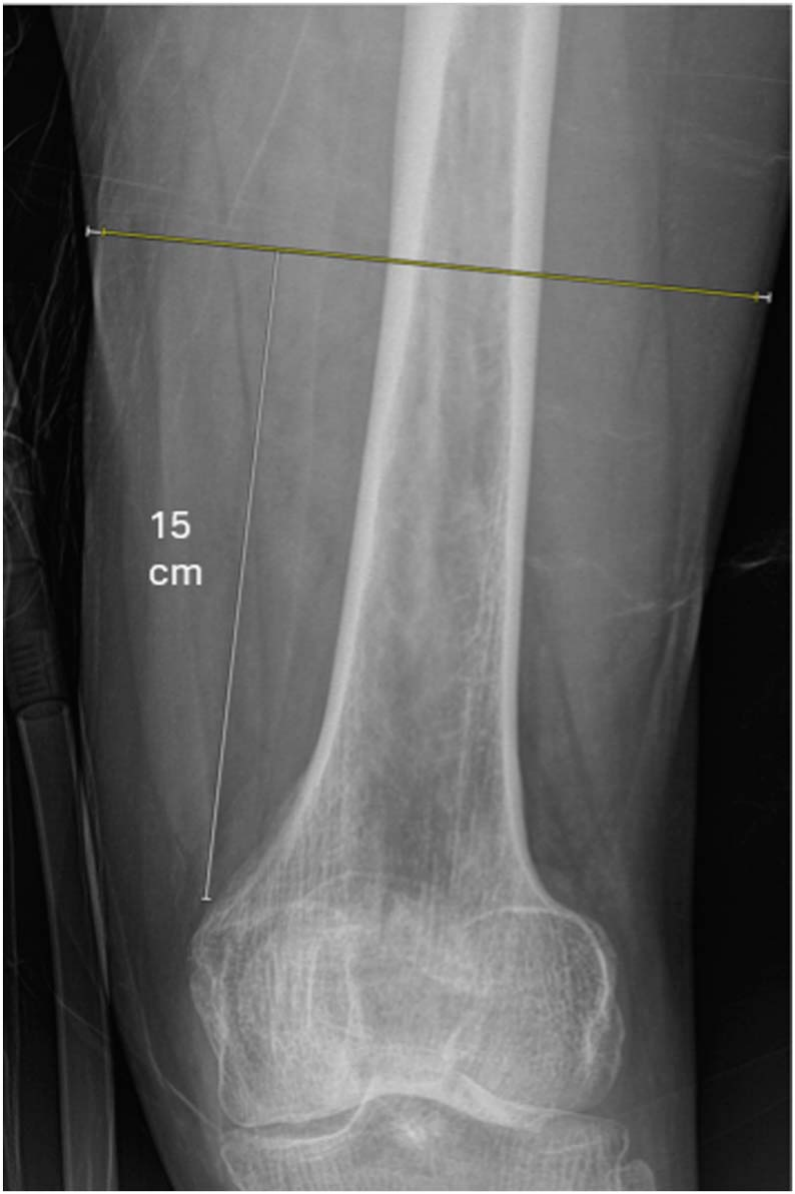
Example of how thigh muscle diameter measurements were obtained from the anteroposterior radiograph. Diameter of thigh muscle (yellow line) and diameter of whole soft tissue envelope (yellow + white lines) were measured 15 cm proximal to the adductor tubercle.

**Fig. 2:**
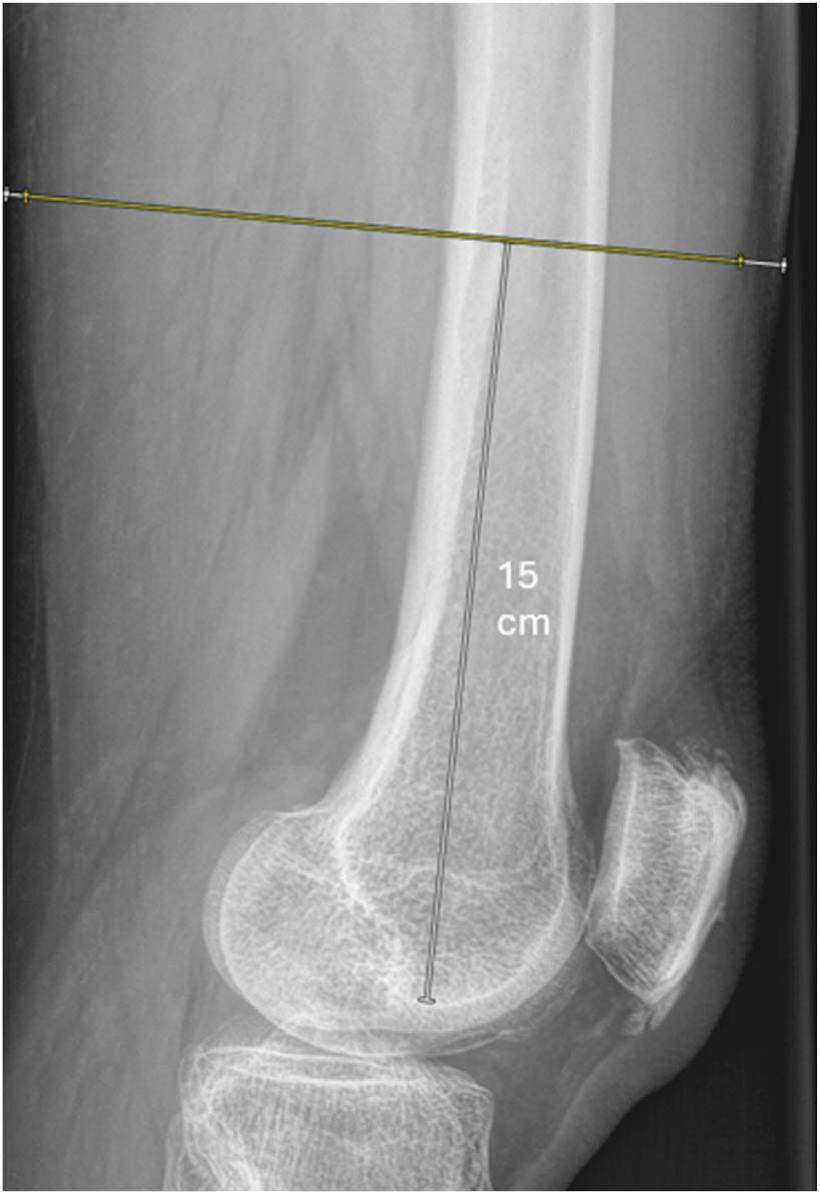
Example of how thigh muscle diameter measurements were obtained from the lateral radiograph. Diameter of thigh muscle (yellow line) and diameter of whole soft tissue envelope (yellow + white lines) were measured 15 cm proximal to the distal terminus of the Blumensaat line.

Radiographic measurements of thigh muscle diameter were determined on radiographs taken during the emergency department admission for the included fracture and before surgery. The specific measurements were adopted from Lurie et al.^11^ Diameter of thigh muscle and diameter of whole soft tissue envelope were measured on the anteroposterior (AP) radiograph 15 cm proximal to the adductor tubercle and on the lateral radiograph 15 cm proximal to the distal terminus of the Blumensaat line (Figs. 1 and 2). Radiographic measurements were performed by 2 musculoskeletal radiology fellows who each assessed half of the data set. A senior musculoskeletal radiologist with 10 years of experience independently assessed 72 measurements to determine inter-rater reliability using intraclass correlation coefficients (ICCs). ICCs were interpreted according to commonly used thresholds: <0.5 = poor, 0.5 to 0.75 = moderate, 0.75 to 0.9 = good, and >0.9 = excellent reliability^17^. For each of the 199 patients, 4 radiographic measurements were planned (n = 796). Overall, 172 of 796 (22%) were not able to be obtained, with reasons for missing measurements including incomplete visualization of the region of interest (n = 129), overlap with radio-opaque material (n = 31), projection artifacts (n = 8), and overlapping soft tissue (n = 4). All radiographic measurements were performed using Visage 7.1 clinical PACS using radiographic diagnostic monitors.

### Explanatory Variables

Baseline characteristics of included patients were obtained from the electronic patient records using chart review. These characteristics included age, sex, race (Asian, Black, Native Hawaiian or other Pacific Islander, White), ethnicity (Hispanic or Latino, or Non-Hispanic or -Latino), prefracture living situation (home, short-term facility, long-term facility), body mass index (BMI, in kg/m^2^), preinjury mobility status (independent, with cane, with walker, wheelchair bound) smoking status (current or former vs. never), presence of dementia, Charlson Comorbidity Index (CCI)^18^, CGA-FI, injury mechanism (same level falls or multilevel falls)^19^, American Society of Anesthesiologists (ASA) physical status classification system^20^, method of anesthesia (general or spinal), fracture location (femoral neck, intertrochanteric, or subtrochanteric), and surgical construct (total hip arthroplasty, hemiarthroplasty, dynamic or sliding hip screw, cephalomedullary nail, or closed reduction and percutaneous pinning). Same-level falls were defined by falls from chair or standing height^19^.

### Outcomes

The primary outcome was 1-year mortality. Mortality data were obtained from the Social Security Index^21^. Secondary outcomes included in-hospital complications (congestive heart failure, pneumonia, urinary tract infection, delirium, urinary retention, and intensive care unit admission). Other collected outcomes included length of stay, discharge disposition, in-hospital mortality, and 30-day mortality.

### Statistical Analysis

Categorical data were represented as frequencies with percentages. Continuous data were represented as means with standard deviation or medians with interquartile ranges, depending on data distribution based on visual inspection of histogram. In bivariate analysis, difference in explanatory variables were compared based on 1-year mortality status using Chi-Squared or Fisher exact tests for categorical variables and *t*-tests or Wilcoxon signed rank tests for continuous variables as appropriate. Univariate logistic regression was performed to determine unadjusted odds ratios with 95% confidence intervals (CIs) for the association between the 4 radiographic measurements and 1-year mortality. Multivariable logistic regression was performed to determine the independent association between the radiographic measurements and 1-year mortality. It was predetermined to include age, sex, CCI, CGA-FI, and BMI as these variables have been previously associated with 1-year mortality^22-25^. In addition, explanatory variables that were marginally associated (p-value <0.10) with 1-year mortality in bivariate analysis were included in the model^26^. The presence of dementia, ASA score, and surgical construct met this threshold but were not included in the final model. Dementia is already captured by both the CCI and the CGA-FI, while ASA classification conceptually overlaps with these indices as a measure of overall comorbidity burden. Surgical construct is typically determined by fracture pattern and patient characteristics. Furthermore, it was hypothesized that BMI may be correlated with the radiographic measurements. However, the Pearson r value was less than 0.70 for all measurement suggesting absence of problematic collinearity (Appendix 2)^27^. For multivariable analysis, missing explanatory variables were imputed using multiple imputation by chained equations, with less than 2% of explanatory variables being missing. Missing radiographic measurements were not imputed. The association between diameter of thigh muscle on AP radiograph and 1-year mortality was visually examined using a locally weighted scatterplot smoothing (LOESS) curve. A post hoc sensitivity analysis was performed to determine the independent association between radiographic thigh measurements normalized for height in meters (thigh measurement to height ratio) and 1-year mortality. For all tests, significance was set at a 2-tailed p of < 0.05. Statistics were performed in R version 4.4.1 (R Foundation for Statistical Computing).

## Results

### Baseline Characteristics

A total of 725 potentially eligible patients were identified. After excluding 469 patients without CGA-FI data, 29 patients with multiple injuries, 27 patients without available imaging, and 1 patient with a nonincludable fracture on imaging review, 199 patients were included in the final analysis (Appendix 3). The median age was 85 years (interquartile range [IQR] 78-90), and 136 (68%) were female (Table I). The median CCI was 4 (IQR 4-7), the mean CGA-FI 0.32 (SD 0.14), the median BMI 23.3 (IQR 20.8-26.9), and 164 patients (82%) lived at home before sustaining the fracture. Most patients (n = 177, 94%) suffered a low mechanism of injury, resulting in a femoral neck (n = 80, 40%), intertrochanteric (n = 109, 55%), or subtrochanteric (n = 10, 5%) fracture.

**TABLE I:** Baseline Characteristics of Included Patients with Bivariate Comparison by 1-Year Mortality

	Total n = 199	Survived n = 155	Deceased n = 44	p
Age in years, median (IQR)	85 (78-90)	84 (78-89)	85 (81-94)	0.022
Sex, n (%)				0.135
Female	136 (68)	110 (71)	26 (61)	
Race, n (%)				0.417
Asian	6 (3)	5 (3)	1 (2)	
Black	6 (3)	3 (2)	3 (7)	
Native Hawaiian or other Pacific Islander	1 (1)	1 (1)	-	
White	177 (93)	138 (94)	39 (91)	
Ethnicity, n (%)				0.687
Hispanic or Latino	9 (5)	8 (5)	1 (2)	
Non-Hispanic/Non-Latino	182 (95)	142 (95)	40 (98)	
Prefracture living situation, n (%)				0.038
Home	164 (82)	133 (86)	31 (70)	
Short-term facility	6 (3)	3 (2)	3 (7)	
Long-term facility	29 (15)	19 (12)	10 (23)	
Body mass index in kg/m^2^, n (%)	23.3 (20.8-26.9)	23.5 (20.8-27.3)	22.7 (21.2-24.8)	0.355
Preinjury mobility status, n (%)				0.147
Independent	75 (38)	62 (40)	13 (30)	
With cane	40 (20)	34 (22)	6 (14)	
With walker	72 (36)	50 (32)	22 (50)	
Wheelchair	11 (6)	8 (5)	3 (7)	
Smoking status, n (%)				0.018
Current or former	95 (48)	67 (44)	28 (64)	
Never	103 (52)	87 (56)	16 (36)	
Dementia, n (%)				<0.001
Yes	21 (11)	9 (6)	12 (27)	
CCI, n (%)	4 (4-7)	4 (4-6)	7 (5-8)	<0.001
CGA-FI, mean (SD)	0.32 (0.14)	0.30 (0.13)	0.41 (0.14)	<0.001
Prefrail, n (%)	42 (21)	41 (26)	1 (2)	
Mild frailty, n (%)	45 (23)	34 (22)	11 (25)	
Moderate frailty, n (%)	48 (24)	41 (26)	7 (16)	
Severe frailty, n (%)	64 (32)	39 (25)	25 (57)	
Injury mechanism, n (%)				0.126
Same level falls	177 (94)	140 (93)	37 (100)	
Multilevel falls	11 (6)	11 (7)	-	
ASA, n (%)				<0.001
≤II	20 (10)	18 (12)	2 (5)	
III	147 (75)	123 (80)	24 (55)	
≥IV	30 (15)	12 (8)	19 (41)	
Method of anesthesia, n (%)				0.589
General	175 (89)	137 (90)	38 (86)	
Spinal	22 (11)	16 (10)	6 (14)	
Fracture location, n (%)				0.473
Femoral neck	80 (40)	60 (39)	20 (45)	
Intertrochanteric	109 (55)	88 (57)	21 (48)	
Subtrochanteric	10 (5)	7 (5)	3 (7)	
Surgical construct, n (%)				0.068
Total hip arthroplasty	7 (4)	7 (5)	-	
Hemiarthroplasty	56 (28)	40 (26)	16 (36)	
Sliding hip screw/Dynamic hip screw	23 (12)	22 (14)	1 (2)	
Cephalomedullary nail	103 (52)	79 (51)	24 (55)	
Closed reduction percutaneous pinning	10 (5)	7 (5)	3 (7)	

ASA = American Society of Anesthesiologists (physical status classification system), CCI = Charlson Comorbidity Index, and CGA-FI = Comprehensive Geriatric Assessment–based Frailty Index.

Missing values for: race (n = 9, 5%); ethnicity (n = 8, 4%); BMI (n = 15, 8%); preinjury mobility status (n = 1, 1%); smoking status (n = 1, 1%); dementia (n = 6, 3%); CCI (n = 6, 3%); injury mechanism (n = 11, 6%); ASA (n = 2, 1%); method of anesthesia (n = 2, 1%).

### Radiographic Measurements

On AP radiographs, the median thigh muscle diameter was 11.78 cm (IQR 10.40-13.72, n = 162), and the median diameter of whole soft tissue envelope was 15.40 cm (IQR 13.40-17.50, n = 142) (Appendix 4). On the lateral radiograph, the median thigh muscle diameter was 12.40 cm (IQR 10.87-14.20, n = 165), and median diameter of whole soft tissue envelope was 15.90 cm (IQR 13.41-17.44, n = 159). The ICCs were good to excellent, with 0.77 for AP thigh, 0.97 for AP soft tissue, 0.90 for lateral thigh, and 0.94 for lateral soft tissue (Appendix 5).

### Outcomes Descriptive

The 1-year mortality rate was 22% (n = 44) (Table II). Specifically, 4 patients (2%) died during hospitalization and 10 patients (5%) died within 30 days of admission. The median length of stay was 5 days (IQR 4-6) with most patients (n = 163, 82%) being discharged to a skilled nursing facility.

**TABLE II:** Outcomes of Included Patients

	Total n = 199
Complications, n (%)	
Congestive Heart Failure	5 (3)
Pneumonia	3 (2)
Urinary Tract Infection	6 (3)
Delirium	37 (19)
Urinary retention	11 (6)
Intensive Care Unit admission	6 (3)
Length of stay, median (IQR)	5 (4-6)
Discharge disposition, n (%)	
Home	22 (11)
Skilled Nursing Facility	163 (82)
Assisted Living Facility	10 (5)
Mortality, n (%)	
In-hospital	4 (2)
30-day	10 (5)
1-year	44 (22)

IQR = interquartile range.

### Multivariable Analysis

Bivariate analysis for 1-year mortality is displayed in Table III. In multivariable analysis, a greater diameter of thigh muscle on the AP radiograph was associated with significantly lower odds of 1-year mortality (odds ratio [OR] 0.74, 95% CI 0.56-0.97, p = 0.028), corresponding to an average 26% reduction in odds of 1-year mortality for each 1 cm increase in thigh muscle diameter, after adjusting for age, sex, smoking status, prefracture living situation, CCI, CGA-FI, and BMI (Table III). A LOESS curve illustrated an approximately linear downward trend in 1-year mortality with increasing thigh muscle diameter, with wider confidence bands at the extremes indicating greater uncertainty in those ranges (Appendix 6). There was no significant association with thigh muscle diameter on lateral radiographs (p = 0.844), or with soft tissue size on AP (p = 0.076) or lateral (p = 0.630) radiographs.

**TABLE III:** Unadjusted and Adjusted Odds Ratios for the Association Between Radiographic Thigh Measurements and 1-Year Mortality

	n	Unadjusted OR (95% CI)	p	Adjusted OR (95% CI)	p
AP radiograph: diameter of thigh muscle	162	0.80 (0.65-0.97)	0.031	0.74 (0.56-0.97)	0.028
AP radiograph: diameter of whole soft tissue envelope	142	0.82 (0.69-0.96)	0.016	0.80 (0.62-1.02)	0.076
Lateral radiograph: diameter of thigh muscle	165	0.91 (0.77-1.08)	0.279	0.90 (0.76-1.25)	0.844
Lateral radiograph: diameter of whole soft tissue envelope	159	0.90 (0.79-1.02)	0.117	0.95 (0.78-1.16)	0.630

AP = anteroposterior, CI = confidence interval, n = number of patients included in analysis, and OR = odds ratio.

Odds ratios are derived from separate logistic regression models for each radiographic parameter. Adjusted odds ratios are adjusted for sex, age, smoking status, prefracture living situation, Charlson Comorbidity Index, Comprehensive Geriatric Assessment–based Frailty Index, and body mass index.

### Post hoc sensitivity analysis

After normalizing the radiographic measurements for height, a greater diameter of thigh muscle on the AP radiograph remained significantly associated with lower odds of 1-year mortality (OR 0.73, 95% CI 0.56-0.96, p = 0.020) while adjusting for age, sex, smoking status, prefracture living situation, CCI, CGA-FI, and BMI (Appendix 7). Furthermore, a greater diameter of whole soft tissue envelope on the AP radiograph became associated with significantly lower odds of 1-year mortality (OR 0.65, 95% CI 0.42-0.98, p = 0.042). There was no significant association between the measurements on the lateral radiographs normalized for height and 1-year mortality.

## Discussion

Given the rising incidence of hip fractures and their associated high 1-year mortality, developing robust methods for prognostication of outcomes is important. Measures of sarcopenia have been previously identified as markers for mortality. However, these may not be feasible for the acute hip fracture setting as they rely on advanced imaging or functional testing^10,28^. This study revealed that a greater thigh muscle diameter on plain AP radiographs obtained at admission to the emergency department was independently associated with reduced odds of 1-year mortality. Specifically, each centimeter increase in thigh muscle diameter was associated with an average reduction in odds of 1-year mortality of 26%. There was no significant association with thigh muscle diameter on lateral radiographs or with soft tissue size on AP or lateral radiographs. In sensitivity analysis normalizing radiographic measurements for height, greater diameter of thigh muscle on the AP radiograph remained significantly associated with lower odds of 1-year mortality.

Sarcopenia is an important driver for adverse outcomes after hip fractures, including functional decline, impaired quality of life, and mortality^6-9^. Sarcopenic patients typically have reduced functional reserve and muscle strength which limits the ability for early recovery after surgery and increases the risk of complications^29,30^. In addition, sarcopenia is known to coexist with protein-energy malnutrition, which diminishes the capacity to withstand surgical stress and accelerates catabolic processes^31^. Therefore, measures of sarcopenia may be helpful for prognostication of outcomes in hip fracture patients. Several studies have used CT-based muscle measurements as a proxy for sarcopenia^32^. Specifically, prior studies demonstrated that lower CT-based muscle mass was independently associated with higher mortality risk following hip fracture surgery^33-38^. However, CT scans are not ubiquitously part of the workup for hip fractures which limits the clinical utility within this context^36^. This study findings demonstrated that thigh muscle diameter on standard AP radiographs obtained at admission was associated with 1-year mortality. This suggests that AP thigh muscle diameter may serve as a helpful adjunct to other known predictors of mortality (e.g., frailty and comorbidities) for more accurate prognostication of mortality. Importantly, thigh muscle diameter on AP radiographs predicts mortality independent on the patients' medical comorbidities and frailty as measured by the CGA-FI. This is an important finding, showing that currently used geriatric assessment tools may not fully capture the overall condition of the patient and adequately predict the risk of complications and mortality.

This study did not find an association with muscle diameter on lateral radiographs and 1-year mortality. This was despite previous studies demonstrating an association between diameter of thigh muscle on lateral radiograph and sarcopenia^11^. One possible explanation is that the positioning required for obtaining a radiograph in suspected hip fracture cases is often suboptimal due to pain. This could have distorted anatomical landmarks and reduced the reliability of muscle or soft tissue measurements on the lateral radiograph. Furthermore, in contrast to the AP radiograph, the muscle is not spread out or flattened on the lateral radiograph. In the AP view, pressure from lying on the radiology board compresses the muscle, altering its shape. This compressive force is absent in the lateral view, which may lead to less consistent muscle measurements and reduced usability. Similarly, soft tissue size on AP and lateral radiographs was not associated with 1-year mortality in multivariable analysis. When normalizing the radiographic measurements for patient height, a greater diameter of whole soft tissue envelope on the AP radiograph was associated with significantly lower odds of 1-year mortality. This may support earlier studies demonstrating that subcutaneous fat in geriatric hip fracture patients is protective for 1-year mortality^39^. However, considering that the whole soft tissue envelope includes the thigh muscle, this association may also be driven by the association of greater thigh muscle size and mortality. Future studies should further determine how radiographic thigh measurements can be best used for prognostication.

The 1-year mortality rate in this study was 22%, which aligns with what has been reported in the current literature, ranging from 22 to 33%. Our complication rates were 2% for pneumonia, 3% for urinary tract infection, and 19% for delirium, compared with reported literature rates of 4%, 7%, and 9.5%, respectively^40,41^. Combined these rates demonstrate the tough prognosis geriatric hip fracture patients are exposed to, highlighting the importance of adequate risk stratification. While some differences in complication and mortality rates were observed, these may have been influenced by our relatively small sample size. This limits the strength of any conclusions regarding the association between radiographic measurements and the occurrence of in-hospital complications and should be kept in mind when applying these findings to broader populations.

There are several limitations that should be considered when interpreting these study findings. First, radiographs were subject to variation as obtaining good quality radiographs can be difficult in patients with hip fractures with factors including limb alignment and fracture hematoma and associated edema potentially influencing measurements. However, the relatively distal location of the radiographic measurements likely minimizes the effects of fracture hematoma and edema, and the cylindrical shape of the soft-tissue envelope of the femur limits the effect of variations in rotation. In addition, radiographs were not calibrated so differences in magnification may have influenced the measurements, although this reflects the real-world conditions. Second, the generalizability of the results may be limited by the strict inclusion and exclusion criteria, which required patients to be at least 70 years old and to have a documented CGA-FI, resulting in the exclusion of a large number of screened patients, and by the fact that radiographs of the distal femur are not routinely obtained in all hip fracture patients. Identifying proxy measures of sarcopenia that can be assessed using standard hip or pelvis radiographs would be more practical for hip fracture patients than relying on measurements taken around the distal femur. Third, this study did not propose a specific cutoff value, as based on this study data and sample size determining individual risks associated with specific ranges of thigh muscle size was deemed premature. Fourth, injury mechanism was not included in the final model since it was not marginally associated with 1-year mortality in this study and none of the deceased patients had sustained multilevel falls.

## Conclusion

Greater thigh muscle diameter measured on anteroposterior radiographs was independently associated with decreased 1-year mortality following hip fracture surgery, with each centimeter increase in diameter being associated with an average reduction in odds of 26%. This should be interpreted in the context of the limited sample size, homogenous patient population, and range of observed thigh muscle sizes. Overall, the results of this study suggest that radiographic parameters may potentially serve to complement currently used modalities, such as frailty assessment, in supporting individualized care.

## Appendix

Supporting material provided by the authors is posted with the online version of this article as a data supplement at jbjs.org (http://links.lww.com/JBJSOA/B12). This content was not copyedited or verified by JBJS.
